# Metaplastic Breast Cancer: A Case Report on a Rare Neoplasm of the Breast

**DOI:** 10.7759/cureus.63717

**Published:** 2024-07-02

**Authors:** Shipha Akanchha Kujur, Deepali Tirkey, Deepanshu Singh, Saurav Banerjee, Chanchal Ashok

**Affiliations:** 1 Pathology, Rajendra Institute of Medical Sciences, Ranchi, IND

**Keywords:** aggressive, case report, triple-negative, metaplastic, breast cancer

## Abstract

Metaplastic breast cancer represents a very rare and histopathologically diverse subtype of breast cancer. It shows neoplastic epithelial differentiation into squamous cells and/or mesenchymal-like components, resulting in its aggressive behavior and poor prognosis compared to other types of breast cancer. Here, we describe the case of a 43-year-old woman diagnosed with metaplastic carcinoma of the breast who presented like any other case of breast lump in the right breast for six months. The tumor had a large size with an ulcerative lesion of the breast. Ultrasound showed heterogeneous echogenicity and lymph node involvement. Surgical resection with axillary lymph node dissection was done. The microscopic examination after tissue processing showed highly pleomorphic tumor cells along with chondromyxoid stroma and osseous differentiation, suggestive of metaplastic breast cancer which was triple-negative for estrogen receptor, progesterone receptor, and human epidermal growth factor receptor 2 on immunohistochemistry. The axillary lymph nodes identified were negative for tumor cells. The rarity and aggressive nature of this cancer pose diagnostic challenges and highlight the importance of multidisciplinary approaches for effective management.

## Introduction

Metaplastic breast cancer (MBC) is an uncommon neoplasm that comprises fewer than 1% of all neoplasms of the breast with histologic and molecular variations, often showing differentiation of neoplastic epithelium into squamous cells and/or mesenchymal-like components [1]. Many studies convey its aggressive behavior and poor prognosis in comparison to other neoplasms of the breast, highlighting its rarity, heterogeneous nature, and absence of treatment therapies [2].

It is characterized by the differentiation of tumor cells into either purely epithelial forms or a combination of epithelial and mesenchymal elements. The epithelial form includes squamous cell carcinoma, adenocarcinoma with spindle cell differentiation, and adenocarcinoma variants such as mucoepidermoid carcinoma. A combination of epithelial and mesenchymal components includes tumors with chondroid or osseous metaplasia, as well as carcinosarcoma.

Managing metaplastic breast cancer poses significant clinical challenges, including understanding its pathogenesis and clinicopathological characteristics and formulating effective treatment strategies [3].

Here, we describe a case involving a young woman diagnosed with metaplastic carcinoma of the breast. The involvement of axillary lymph nodes is less common as it tends to spread more through the hematogenous route rather than the lymphatic system. Metaplastic breast cancers are typically triple-negative, although they can sometimes exhibit positivity for hormone receptors and human epidermal growth factor receptor 2 (HER2).

## Case presentation

A 43-year-old woman presented to the Department of Surgery, Rajendra Institute of Medical Sciences, Ranchi with the complaint of a lump in her right breast for six months. She had no family history of breast cancer. Her medical history revealed a lump in the right breast which was painless and gradually increasing in size over the past six months. Approximately two months before the presentation, the lump had ulcerated. Initial physical examination indicated a firm lump measuring approximately 9 × 5.7 cm with ulceration and everted edges, not attached to the chest wall, and palpable ipsilateral lymph nodes of the axilla.

Ultrasonography showed a heterogenous mass measuring approximately 10 × 6 cm encompassing all quadrants of the breast, displaying posterior acoustic shadowing along with increased vascularity and dilated ducts extending from the mass to the nipple (classified as Breast Imaging-Reporting and Data System 5). Additionally, ultrasound imaging detected a few enlarged lymph nodes with thickened cortex, some exhibiting a loss of fatty hilum, suggestive of potential metastasis.

A core needle biopsy from the ulcer margin revealed predominantly oval to spindle-shaped cells organized in a diffuse sheet. These cells exhibited oval-to-spindle-shaped hyperchromatic nuclei with prominent eosinophilic nucleoli along with abundant cytoplasm. Additionally, a few tumor giant cells were observed.

**Figure 1:**
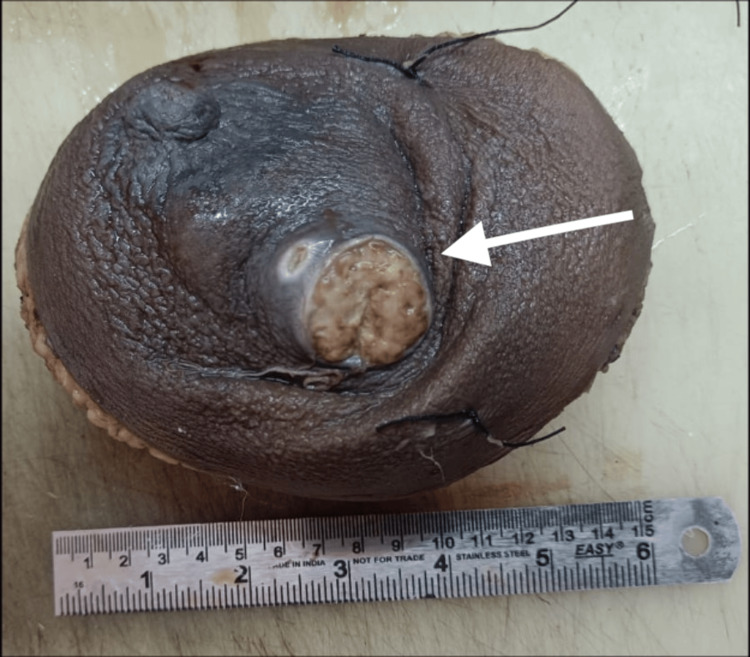
An ulcer (white arrow) in the upper outer quadrant of the right breast (gross specimen).

The patient was planned and posted for modified radical mastectomy. Thereafter, we received the right breast specimen in the Department of Pathology, RIMS, Ranchi for the histopathological examination. Grossly, the specimen measured approximately 16 × 12 × 6 cm. An ulcer was noted in the upper outer quadrant measuring approximately 4 × 3.5 cm (Figure 1).

**Figure 2:**
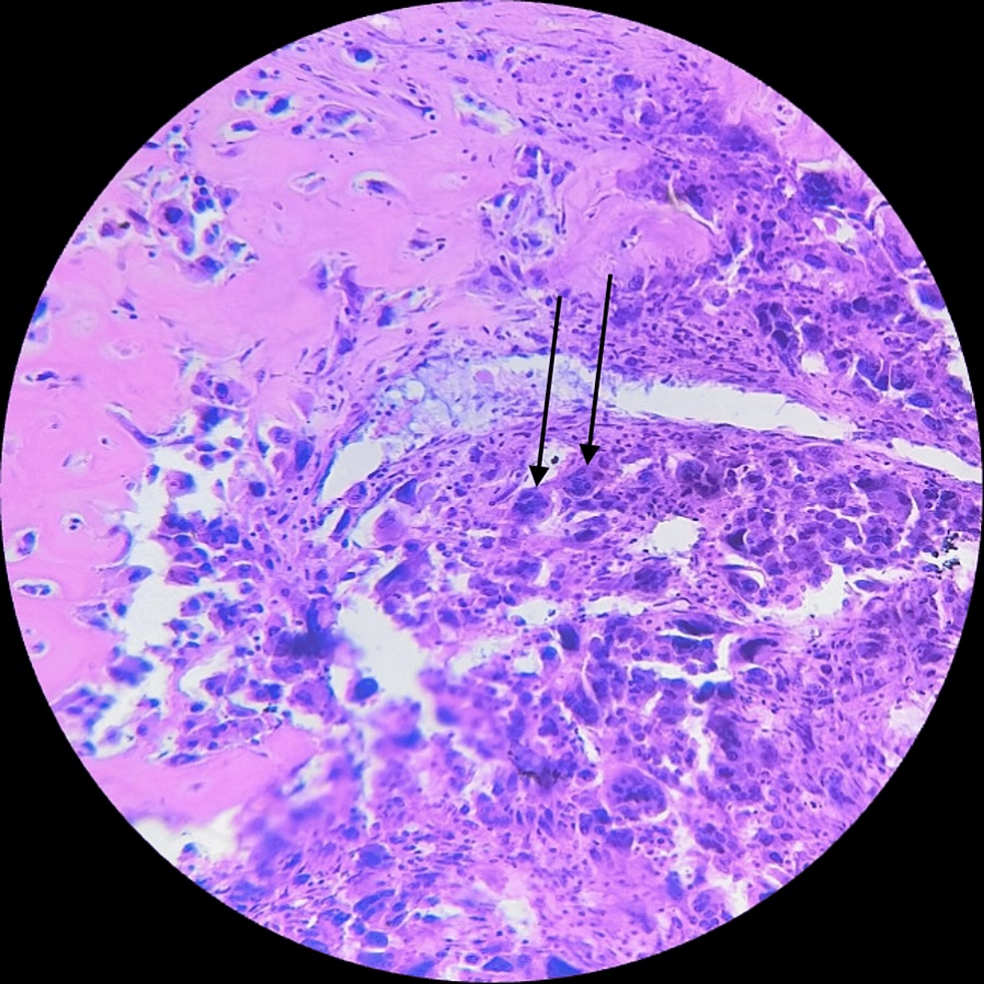
Section showing highly pleomorphic tumor cells with a fair number of tumor giant cells (black arrow) and brisk mitotic activity with the nucleus showing dispersed chromatin and prominent nucleoli (high magnification; 40×).

**Figure 3:**
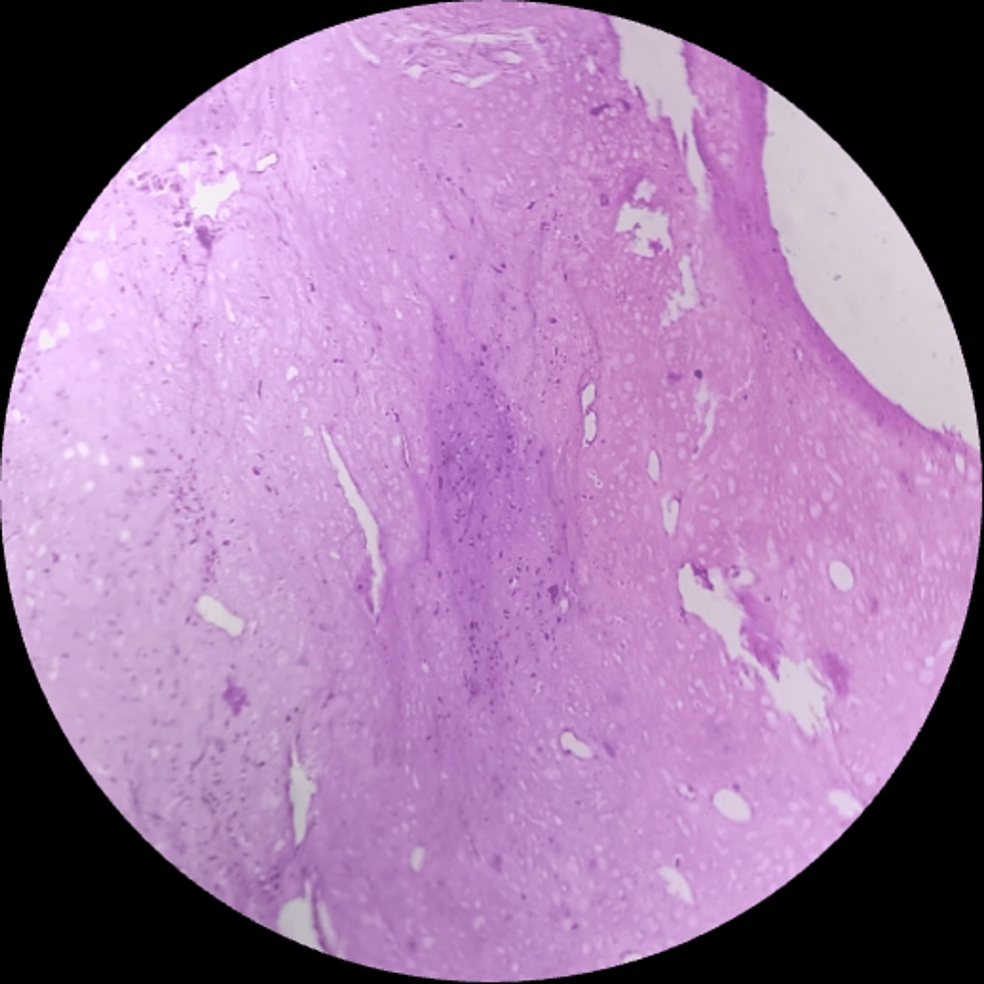
Section showing chondromyxoid stroma (low magnification; 10×).

**Figure 4:**
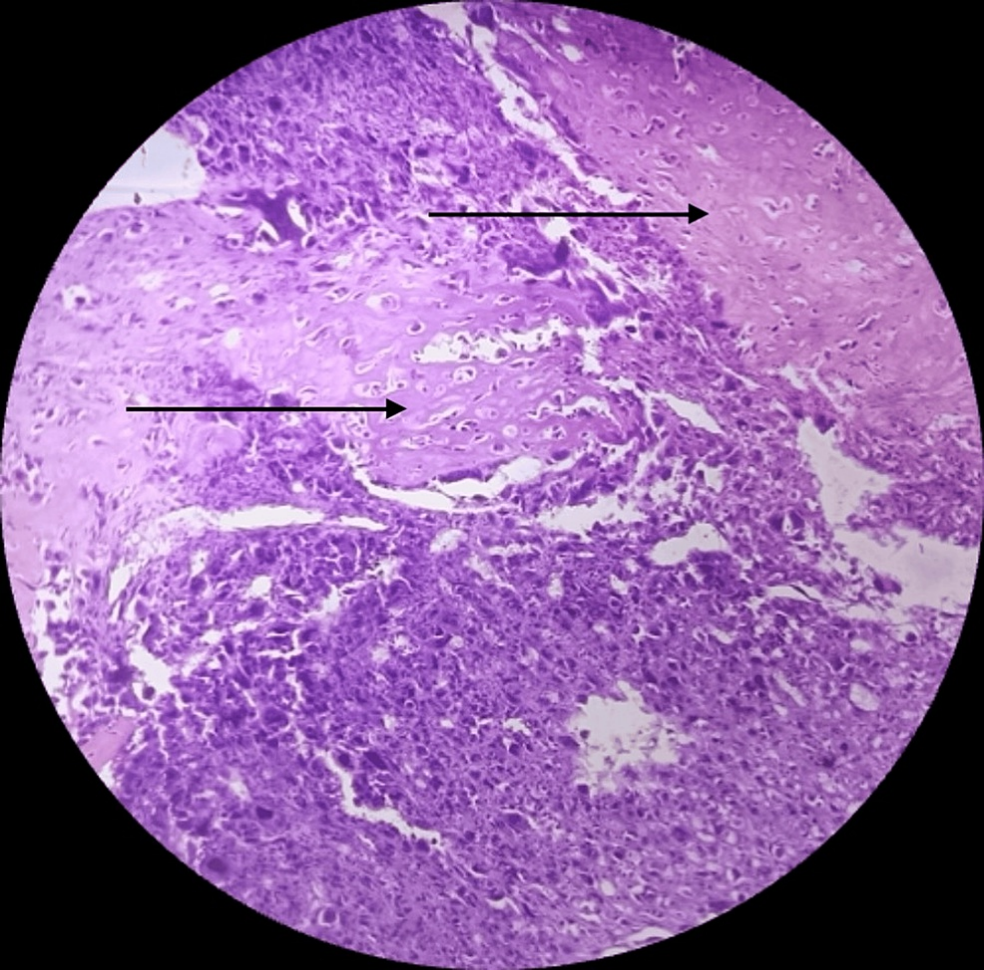
Section showing osseous differentiation (black arrows) (high magnification; 40×).

**Figure 5:**
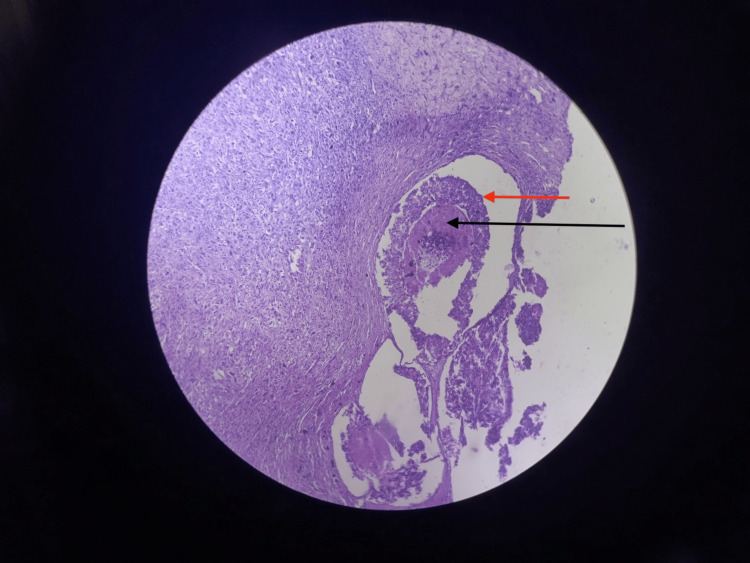
Section showing areas of necrosis (black arrow) and ductal carcinoma in-situ component (red arrow) (low magnification; 10×).

**Figure 6:**
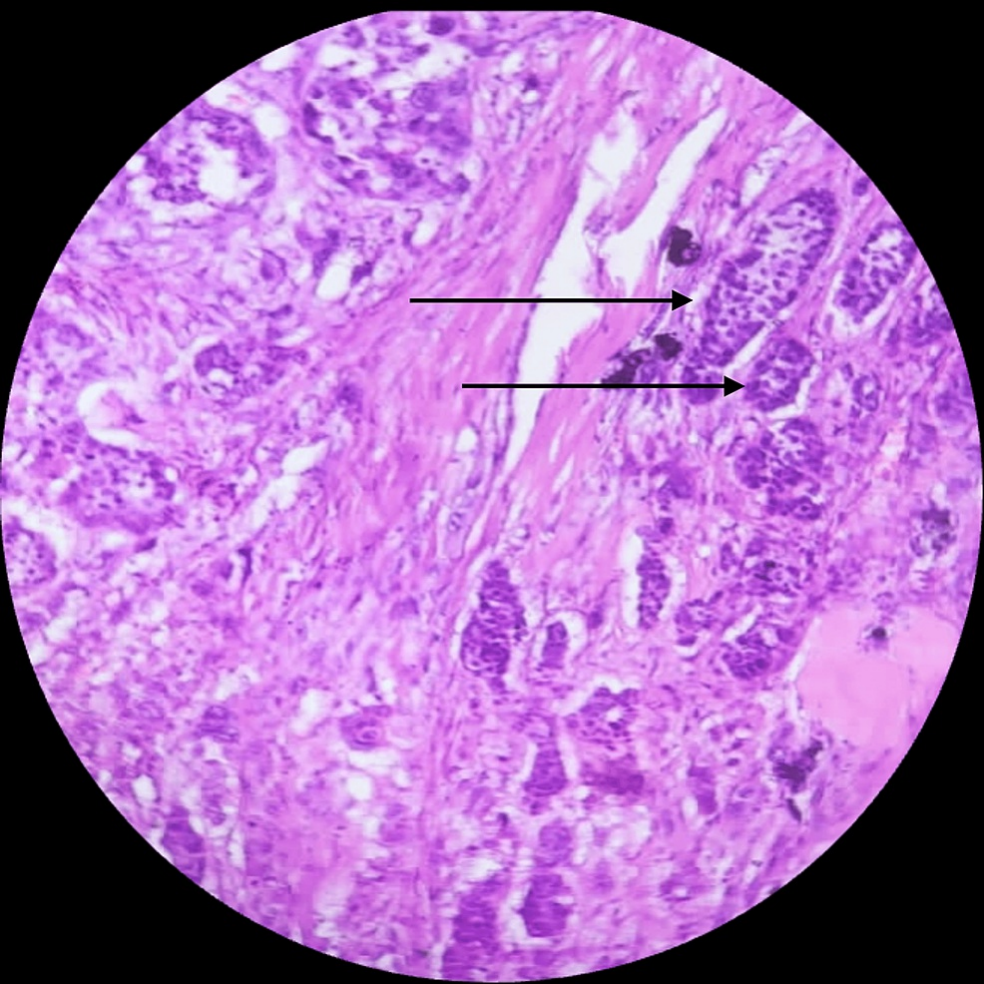
Section showing tumor cells (epithelial component) arranged in a nested pattern (black arrows) (high Magnification; 40×).

Histologically, the examined sections revealed highly pleomorphic tumor cells with a fair number of tumor giant cells, and brisk mitotic activity was also seen with the nucleus showing dispersed chromatin and prominent nucleoli (Figure 2) with chondromyxoid stroma (Figure 3) and osseous differentiation at some places (Figure 4). Tumor cells were round, oval, and spindle-shaped with indistinct cytoplasm. Widespread areas of necrosis and ductal carcinoma in-situ components were also seen (Figure 5). Some places showed tumor cells (epithelial components) arranged in a nested pattern (Figure 6).

The sample was negative for estrogen receptor, progesterone receptor, and HER2. A total of 12 lymph nodes were identified but all were negative for tumor cells. Lymphovascular invasion was absent. Histopathological examination was in favor of metaplastic carcinoma of the right breast (TNM staging: T4N0MX; modified Bloom-Richardson score: 7/9; Grade 2 (moderately differentiated)). The patient is clinically stable at present. The next follow-up is scheduled after two months.

## Discussion

Metaplastic breast cancer shows a variety of patterns of metaplasia and differentiation along multiple cell lines. The different subgroups of metaplastic breast cancer have shown documented cumulative five-year survival rates of 64%, 49%, 63%, and 68% for spindle cell carcinoma, carcinosarcoma, pure ductal squamous carcinoma, and matrix-producing metaplastic carcinoma, respectively [4]. Similarly, histopathologically, we could appreciate chondroid, osseous, osteogenic giant cell, and epithelial differentiation in our case. Grossly, it presented as another stereotype of breast cancer.

Lungs are the most frequent site of distant metastasis in these cases [5]. Lymph node metastasis is infrequent in primary breast sarcoma as seen in our case where 12 lymph nodes were identified but none showed tumor cell invasion. The clinical and radiological presentation of this condition resembles that of other types of breast cancer. Carter et al. documented the presenting age group of the tumor to be 40 to 96 years, while another study documented it to be 22 to 91 years [5]. Our patient was 43 years old.

Metaplastic breast carcinoma shows positivity for high-molecular-weight cytokeratins (HMWCKs)/basal cytokeratin including CK5/6 and 34βE12 [6]. Another very useful marker with 86.7% sensitivity and 99.4% specificity is p63 which can stain both epithelial and spindle cell components [6]. Expression of CD10 is seen commonly in spindle cell variants [6]. CK7 is expressed in 30% to 60% of metaplastic carcinoma [6].

The potential diagnoses cover a broad spectrum, including phyllodes tumor (negative for HMWCK and CK5/6), primary breast sarcoma (lacking epithelial elements, negative for AE1/AE3, HMWCK, CK5/6, and focal or negative for p63), metastatic sarcoma (negative for AE1/AE3, HMWCK, CK5/6, and focal or negative for p63), adenomyoepithelioma (characterized by a bland epithelial and stromal component and absence of squamous differentiation), myoepithelial carcinoma (composed entirely or predominantly of malignant spindle cells with myoepithelial differentiation and no squamous or other mesenchymal differentiation), and pleomorphic adenoma (typically located in the retroareolar region, featuring a well-organized biphasic pattern with an inner layer of regular epithelial cells, a thin and continuous outer layer of myoepithelial cells, and lacking cytologic atypia, atypical mitosis, or necrosis) [7].

Hence, we emphasize that there is a need for a combination of several stains to be performed (such as cytokeratin cocktail, p63, high-molecular-weight cytokeratins) to make an accurate diagnosis.

## Conclusions

The rarity of metaplastic carcinoma coupled with its unfavorable prognosis underscores the importance of maintaining a high index of suspicion when evaluating a rapidly growing breast lump. Currently, due to the lack of specific treatment guidelines for metaplastic carcinoma, the literature review suggests approaching its management similarly to other types of invasive carcinoma. Given its limited responsiveness to conventional chemotherapy, the potential efficacy of targeted therapy, under investigation in numerous studies, holds promise for improving patient outcomes in the foreseeable future. A self-breast examination should be encouraged to ensure early diagnosis and management of the disease.
